# Genomic Analyses of *Penicillium* Species Have Revealed Patulin and Citrinin Gene Clusters and Novel Loci Involved in Oxylipin Production

**DOI:** 10.3390/jof7090743

**Published:** 2021-09-09

**Authors:** Guohua Yin, Hui Zhao, Kayla K. Pennerman, Wayne M. Jurick, Maojie Fu, Lijing Bu, Anping Guo, Joan W. Bennett

**Affiliations:** 1 Hainan Key Laboratory for Biosafety Monitoring and Molecular Breeding in Off-Season Reproduction Regions, Institute of Tropical Bioscience and Biotechnology, Chinese Academy of Tropical Agricultural Sciences, Haikou 571101, China; zhaohui@itbb.org.cn (H.Z.); maojie_fu8@163.com (M.F.); 2 Department of Plant Biology and Pathology, Rutgers, The State University of New Jersey, New Brunswick, NJ 08901, USA; profmycogirl@yahoo.com; 3 Toxicology and Mycotoxin Research Unit, U.S. Department of Agriculture, Agricultural Research Service (USDA ARS), Athens, GA 30605, USA; kkpennerman@gmail.com; 4 Food Quality Laboratory, U.S. Department of Agriculture, Agricultural Research Service (USDA ARS), Beltsville, MD 20705, USA; wayne.jurick@usda.gov; 5 Center for Evolutionary & Theoretical Immunology (CETI), Department of Biology, University of New Mexico, Albuquerque, NM 87131, USA; xunshengbu@gmail.com

**Keywords:** *Penicillium expansum*, *Penicillium crustosum*, *Penicillium maximae*, genomic analyses, lipoxygenase, mycotoxin, volatile organic compounds

## Abstract

Blue mold of apple is caused by several different *Penicillium* species, among which *P. expansum* and *P. solitum* are the most frequently isolated. *P. expansum* is the most aggressive species, and *P. solitum* is very weak when infecting apple fruit during storage. In this study, we report complete genomic analyses of three different *Penicillium* species: *P. expansum* R21 and *P. crustosum* NJ1, isolated from stored apple fruit; and *P*. *maximae* 113, isolated in 2013 from a flooded home in New Jersey, USA, in the aftermath of Hurricane Sandy. Patulin and citrinin gene cluster analyses explained the lack of patulin production in NJ1 compared to R21 and lack of citrinin production in all three strains. A *Drosophila* bioassay demonstrated that volatiles emitted by *P*. *solitum* SA and *P*. *polonicum* RS1 were more toxic than those from *P*. *expansum* and *P. crustosum* strains (R27, R11, R21, G10, and R19). The toxicity was hypothesized to be related to production of eight-carbon oxylipins. Putative lipoxygenase genes were identified in *P*. *expansum* and *P*. *maximae* strains, but not in *P*. *crustosum*. Our data will provide a better understanding of *Penicillium* spp. complex secondary metabolic capabilities, especially concerning the genetic bases of mycotoxins and toxic VOCs.

## 1. Introduction

As one of the largest and most ubiquitous fungal genera, *Penicillium* currently contains 483 accepted species with varying lifestyles that include saprobes, pathogens, and endophytes capable of growing in a wide range of habitats [1,2,3]. The penicillia are commonly known as blue or green molds because of the distinctive color that the spores of many species give to their macroscopic colonies, easily visible to the naked eye. A large study of buildings and outdoor environments in different seasons and regions in the United States demonstrated that *Penicillium* spp. ranked amongst the most common airborne fungi [4]. The best-known species in the genus have positive economic impacts and include *Penicillium chrysogenum* (formerly *P*. *notatum*), which produces the famous antibiotic penicillin [5]; *P*. *griseofulvum,* which produces the antifungal drug griseofulvin [6]; and *P*. *camemberti* and *P*. *roqueforti,* which are important in cheese fermentation [7].

Many other *Penicillium* species have negative economic effects. In addition to causing allergies in humans [8], these psychrophilic fungal pathogens are the most common causes of spoilage of stored fruits and vegetables and capable of growing at very cold temperatures. Furthermore, many species produce mycotoxins on the various substrates including foods intended for human consumption [9]. For example, *P. italicum* and *P. digitatum* are economically impactful pathogens of citrus fruit, while *P. expansum* decays apples and produces patulin, a toxic secondary metabolite [10,11,12,13]. Other *Penicillium* species are exceptional causes of biodeterioration, especially on water-logged building materials, walls and wallpaper, floors, carpets, mattresses, and upholstered furniture such as those found after hurricane events, and can be used as indicator organisms for indoor dampness [14,15].

Through reduced price and higher throughputs, next-generation sequencing technology has advanced fungal genomic research, especially on fungal secondary metabolite biosynthesis [16]. Many *Penicillium* genomes including *P*. *chrysogenum* [5], *P*. *digitatum* [17,18] *P. expansum* [12,13,19], *P. solitum* [20], *P. roqueforti* [21], *P. griseofulvum* [22], and *P. marneffei* [23] have been sequenced, assembled, and annotated. For a summary of 30 *Penicillium* genomes from 18 species and a complete review of the diversity and mechanisms of genomic adaption, see Ropars et al. [24].

Blue mold of apples can be caused by many different *Penicillium* species, among which *P. expansum* and *P. solitum* are usually considered to be the most important species [25]. Nevertheless, recent studies show that *P. crustosum* can also cause blue mold decay of pome and stone fruits and produce penitrem A, roquefortine C, terrestric acid, and cyclopenol [26]. *P*. *expansum* regularly occurs on apples, pears, and quince [27,28]. In addition to pome fruits, this species also has been reported to cause bulb rot on *Lilium* in India [29], postharvest decay on stored kiwifruit (*Actinidia argute*) in China [30], and blue mold in fresh date palm fruit (*Phoenix dactylifera*) in Spain [31]. Other *Penicillium* species able to cause similar decay include *P. italicum*, *P. digitatum*, *P. commune*, *P. verrucosum*, *P. rugulosum*, *P. polonicum,* and *P. chrysogenum* [25,32,33,34,35]. Of particular concern is the fact that *P*. *expansum* produces patulin, a potential carcinogenic mycotoxin that can contaminate fruit juices, compotes, and other processed apple products [36]. A recent review presented the detailed biosynthetic mechanisms used for secondary metabolite production in *P*. *expansum*, especially focused on patulin biosynthesis [37,38].

*Penicillium* species not only produce mycotoxins but also a variety of volatile organic compounds (VOCs) [39]. In particular, the eight-carbon oxylipin volatile 1-octen-3-ol is very toxic in a *Drosophila* eclosion bioassay [14,40,41]. In order to explore the possible fungal virulence factors and to devise novel strategies to mitigate blue mold contamination in stored fruits, several genomes of *Penicillium* species (*P*. *expansum* R19 and R21, *P*. *polonicum* RS1 and *P*. *crustosum* NJ1) have been reported from our group [19,42,43,44]. In this study, we further analyzed the genomes of *P*. *expansum* R21, *P*. *crustosum* NJ1, and *P. maximae* P113, comparing them with other *Penicillium* and *Aspergillus* species with sequenced genomes. Our long-term goal is to use genomic analyses to elucidate the toxicity of targeted mycotoxins and volatile organic compounds produced by *Penicillium* species. Our specific aims were: (1) to use Illumina sequencing technology to characterize the complete genomes of two *Penicillium* species (*P*. *expansum* R21 and *P*. *crustosum* NJ1) isolated from infected pome fruits, and one *Penicillium* species (*P*. *maximae* 113) isolated from an environmental sample; (2) to compare the mycotoxin gene clusters from these three species with other *Penicillium* and *Aspergillus* spp.; (3) to analyze the toxigenic potential of VOCs from seven *Penicillium* strains including four *P*. *expansum* strains (R11, R19, R21, and R27), one *P. crustosum* (G10), one *P*. *polonicum* (RS1), and one *P*. *solitum* (Stanley A, labeled as SA), in a *Drosophila* bioassay; and (4) to identify the lipoxygenase genes involved in VOCs production from *P*. *expansum*, *P*. *crustosum*, and *P*. *maximae*.

## 2. Materials and Methods

### 2.1. Fungal Preparation and Sequencing

*P*. *expansum* R21 (labeled as R21) was isolated from red delicious apple fruit in Pennsylvania in 2011 and deposited in NCBI with accession number MJGF02000000 [42]; *P*. *solitum* NJ1 (labeled as NJ1) was a subculture of *Penicillium* strain NRRL2159A, renamed as *P*. *crustosum* and deposited in NCBI with accession number MJCB01000000 [45,46]; *P*. *sclerotiorum* 113 (labeled as 113) was a strain isolated from a home in Manasquan, NJ, flooded with marine water after Hurricane Sandy in 2013, renamed as *P*. *maximae* and deposited in NCBI with accession number MJCA01000000 [47,48]. Fresh spores (final concentration at 10^4^/mL) of these three strains were inoculated in potato dextrose broth and incubated with shaking at 200 rpm at 25 °C for seven days in the dark. The total genomic DNA was extracted using the E.Z.N.A. ^®^fungal DNA mini kit (Omega Bio-Tek Inc., Norcross, GA, USA). Sequencing was performed using an Illumina MiSeq Benchtop Sequencer (Illumina, Shanghai, China). The trimming of raw data followed the approach by Yang et al. [49]. SOAPdenovo v2.04 (http://soap.genomics.org.cn, accessed on 6 August 2016) was used to assemble the *Penicillium* spp. genomes. The de novo assembly software ABYSS [50] and reference-guided assembly improvement tool AlignGraph [51] were also used to confirm and further refine the assembly (accessed on 15 August 2016). GapCloser V1.12 was used to fill local gaps and perform base correction [52]. RepeatMasker version 4.0.3 was used to identify and classify the repetitive elements in the genome, and “N” was used to shield the low-complexity sequences and interspersed repeats [53]. Marker 2 was used to perform gene prediction [54].

### 2.2. Bioinformatic Analyses of Penicillium spp.

Using BLAST 2.2.28+ [55], the protein sequences of the predicted genes were aligned with NCBI nr, Genes, STRING, and GO databases to obtain the gene annotation information. COG annotations (Clusters of Orthologous Groups of proteins, http://www.ncbi.nlm.nih.gov/COG/, accessed on 20 August 2016) were obtained by alignment with STRING (v 9.05, http://string-db.org/, accessed on 20 August 2016) database. GO annotation (Gene Ontology, http://www.geneontology.org/, accessed on 20 August 2016) was obtained using Blast2GO software [56]. Homologous genes were identified and analyzed using OrthoMCL (http://www.orthomcl.org/cgi-bin/OrthoMclWeb.cgi, accessed on 20 August 2016). Single copy homologous genes were aligned using MAFFT (http://mafft.cbrc.jp/alignment/software/, accessed on 20 August 2016), and phylogenetic analysis was performed using MEGA (http://www.megasoftware.net/, accessed on 20 August 2016). Secondary metabolite loci of *Penicillium* species were identified using antiSMASH version 3.0.5. [57]. Using the *P. expansum* ATCC 24692 as a reference genome, we identified the patulin gene clusters of *P*. *polonicum* RS1, *P*. *crustosum* NJ1, and *P*. *expansum* strains R21 and R19. Functional conservation was measured with TBLASTN identity using translated protein sequences of reference patulin and citrinin gene clusters as queries aligned against the genome sequences of the selected *Penicillium* and *Aspergillus* strains. Unless otherwise specified, files were written and edited as necessary using in-house Python and Linux scripts. Bioinformatics program and function settings were left at default.

The genomes for *P. maximae* 113 (assembly GCA_001750025.1), *P. expansum* R19 (assembly GCA_000688875.1), *P.* *expansum* R21 (assembly GCA_001750045.2), *P. crustosum* NJ1 (assembly GCA_001750005.1), and *P.* *polonicum* RS1 (assembly GCA_000952775.2) were retrieved from NCBI Genome database (Bethesda, available online: http://www.ncbi.nlm.nih.gov/genome (accessed on 25 April 2017)). Putative *Penicillium* genes were automatically predicted using GeneMark-ES for fungal genomes [58]. The GTF files were converted to multiple FASTA nucleotide sequences using the function gffread (http://github.com/gpertea/gffread, accessed on 25 April 2017) [59]. Gene nucleotides were translated into amino acid sequences according to the NCBI genetic code 4 [60].

Amino acid sequences of fungal lipoxygenase genes were retrieved from NCBI GenBank using the search term “lipoxygenase + ascomycota”. All of the proteins were aligned using Clustal Omega [61]. A subset of proteins specifically annotated as manganese lipoxygenases were separately subjected to multiple sequence alignment. Hidden Markov model profiles were generated for the multiple sequence alignments using the hmmbuild function of HMMER [62]. These profiles were then searched against the *Penicillium* predicted proteomes using the hmmsearch function of HMMER. Lipoxygenases pulled from the searches and genes in the surrounding areas (two genes up and downstream on the same strand) were annotated using Blast2GO [56].

### 2.3. Exposure of Drosophila 3rd Instar Larvae to VOCs Emitted by Seven Penicillium Species

Four *P*. *expansum* strains (R11, R19, R21, and R27), one *P. crustosum* (G10) [63], one *P*. *solitum* strain (SA), and one *P*. *polonicum* RS1 were chosen for the toxicity analysis of VOCs emitted by fungi. As the toxicity of VOCs emitted by *P. maximae* 113 (originally identified as *P*. *sclerotiorum*) has been analyzed in our previous publication [14], it was not included here. For each strain, a 5 mm diameter plug of potato dextrose agar (PDA) was used as inoculum and cultivated for 5 days at 25 °C in the dark on 100 mm × 15 mm Petri dish. White-eyed *Drosophila* flies (*y^1^w^1118^*) with a wild-type immune system were maintained on Ward’s Instant *Drosophila* medium (WARD’s Natural Science) before exposure of 3rd instar larvae using a double petri plate method, as previously described [14]. Fifteen larvae of similar sizes were placed on the larva-pupae medium before attachment to the petri plate with the fungal culture. The experiment was conducted with three replicates in duplicate (six replicates, N = 90) for each *Penicillium* strain. Control larvae were exposed to PDA medium without any fungi. All the double petri plates were incubated at 25 °C with rotation at 50 rpm for 12 days. The numbers of larvae, pupae, adult, and dead flies were counted daily. Student’s *t*-test was used to determine statistical significance of the differences in metamorphic stages between controls and VOC-exposed strains on the 4th day for the larvae, the 7th day for the pupal stage, and the 12th day for the adult and dead flies.

## 3. Results

### 3.1. Fungal Isolation, Sequencing Data Statistics, Genome Assembly, and Gene Prediction

**Figure 1:**
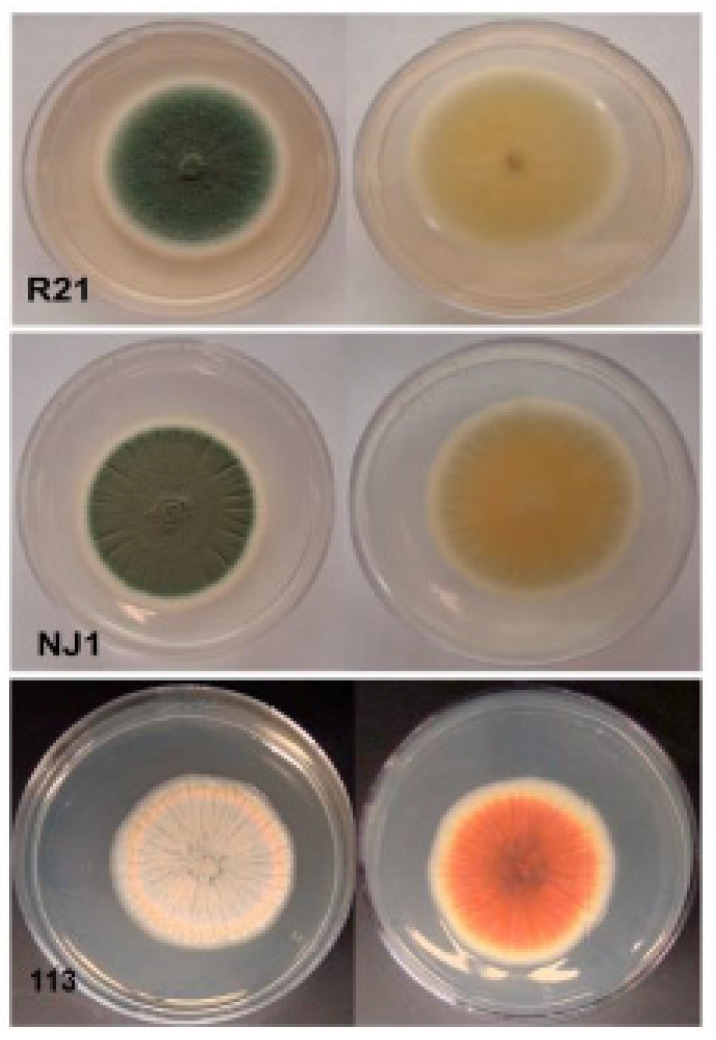
Morphology of *P*. *expansum* R21, *P*. *crustosum* NJ1, and *P*. *maximae* 113 colonies after cultivation for seven days on PDA at 25 °C in the dark on the forward (**left**) and reverse (**right**) sides.

We selected three *Penicillium* species: *P*. *expansum* R21, *P*. *crustosum* NJ1, and *P*. *maximae* 113. For each strain, a 5 mm diameter plug of potato dextrose agar (PDA) was used as inoculum and cultivated for 7 days at 25 °C in the dark on 60 mm × 15 mm petri dish. The sizes, colors, and shapes of these three fungi after seven-day cultivation on PDA are shown in Figure 1. The insert sizes, read length, total bases, Q20%, and average depth are summarized in Table S1. Over 86% of the reads were high quality, and average sequencing depths of the three libraries were also above 67X. After trimming the raw sequencing data according to the approach in our previous study [49], the high-quality sequencing data still had genome coverages of at least 57X. We performed k-mer analyses using the 400–500 bp paired-end libraries to estimate the *Penicillium* spp. genomic sizes to be 32–35 Mb (Table S2). The total lengths of repeated sequences were 263–366 kb, contributing to 0.8–1.15% of the whole genomes (Table S3). Scaffolds assembled de novo and trimmed were used to determine the total number of predicted genes, gene lengths, and GC content (Table 1).

### 3.2. COG Analyses and GO Annotation

Clusters of Orthologous Groups (COGs) were constructed based on the comparisons of proteins from already sequenced genomes. COG database selected the main phylogenetic lineages, and each COG family consists of the proteins from at least three species. The carbohydrate transport and metabolism (G) had the most genes followed by predictions of general function (R), amino acid transport and metabolism (E), energy production and conversion (C), and secondary metabolites biosynthesis, transport, and catabolism (Q). The cell motility (N), extracellular structures (W), and nuclear structure (Y) had the fewest numbers of genes (Figure S1A,C,E). Taking 113 as an example, the predicted genes were annotated as a wide range of functional categories. The three most populous descriptive functional classes were “posttranslation modification, protein turnover, chaperones” (O, 427 genes), “secondary metabolites biosynthesis, transport and catabolism” (Q, 385 genes), and “energy production and conversion” (C, 372 genes). The fewest genes were annotated as being involved in “cell motility” (N, 2 genes) and extracellular structures (W, 4 genes). The category “general functional predictions only” (R) included the 751 genes that could not be functionally annotated (Figure S1E). In all three *Penicillium* strains, more than 655 putative genes were assigned to GO annotations; seven biological processes: cellular process, single-organism process, metabolic process, biological regulation, regulation of biological process, establishment of localization, and localization; two molecular functions: binding and catalytic activity; and six cellular components: organelle, cell compartment, membrane compartment, membrane, macromolecular complex, and cell (Figure S1B,D,F).

### 3.3. Homologous Genes and Phylogenetic Analysis

**Figure 2:**
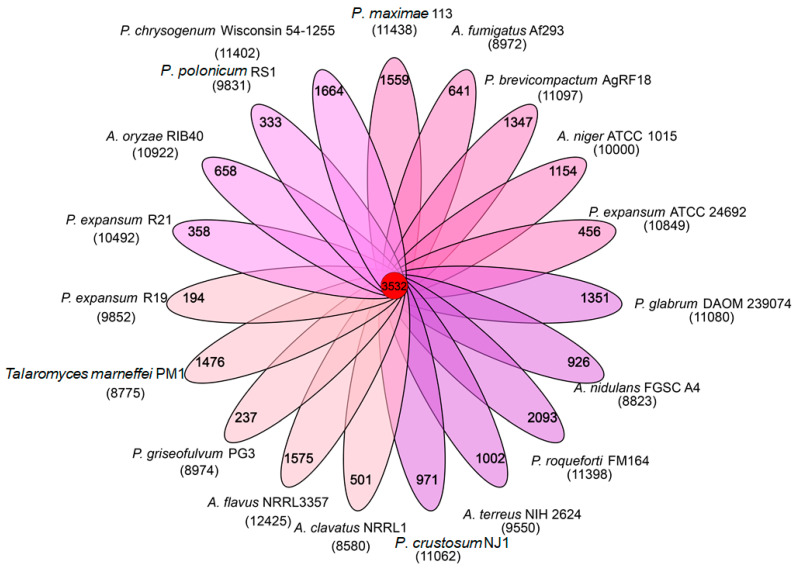
A Venn diagram of the homologous genes from eighteen genomes of *Penicillium* and *Aspergillus* species. Twelve *Penicillium* and seven *Aspergillus* strains were used in the analysis.

Nineteen genomes were chosen for analysis based on the relative detailed information and available genomes, and these filamentous fungal species shared 3532 common homologs. *P*. *roqueforti* FM164 had the highest number of non-homologous genes (2093), followed by *P*. *chrysogenum* Wisconsin 54-1255 (1664), *Aspergillus flavus* NRRL3357 (1575), 113 (1559), *P*. *marneffei* PM1 (renamed as *Talaromyces marneffei*, 1476) [3], and *P*. *glabrum* DAOM 239074 (1351). *P*. *expansum* R19 and *P*. *griseofulvum* PG3 had the fewer number of non-homologous genes, 194 and 237, respectively (Figure 2).

**Figure 3:**
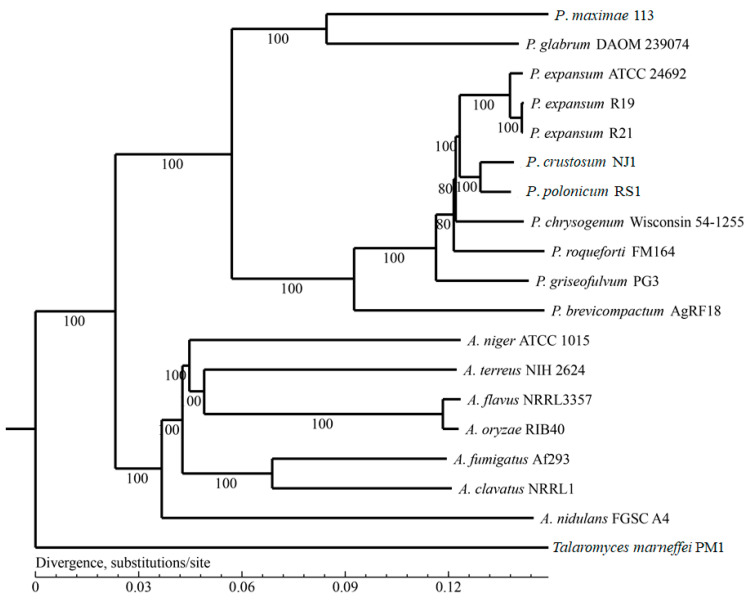
Phylogenetic relationships of selected *Penicillium* and *Aspergillus* species.

Based on the results from the homologous gene analysis, single-copy genes were selected for phylogenomic analysis. Three *P. expansum* strains (ATCC 24692, R19, and R21), *P.* *polonicum* RS1, and *P*. *crustosum* NJ1 strains were closely grouped into their own clades; 113 and *P*. *glabrum* DAOM 239074 were relatively distant from other strains. All seven *Aspergillus* species (ATCC1015, NIH2624, NRRL3357, RIB40, Af293, NRRL1, and FGSC A4) also closely grouped together (Figure 3).

### 3.4. Patulin and Citrinin Gene Clusters

**Figure 4:**
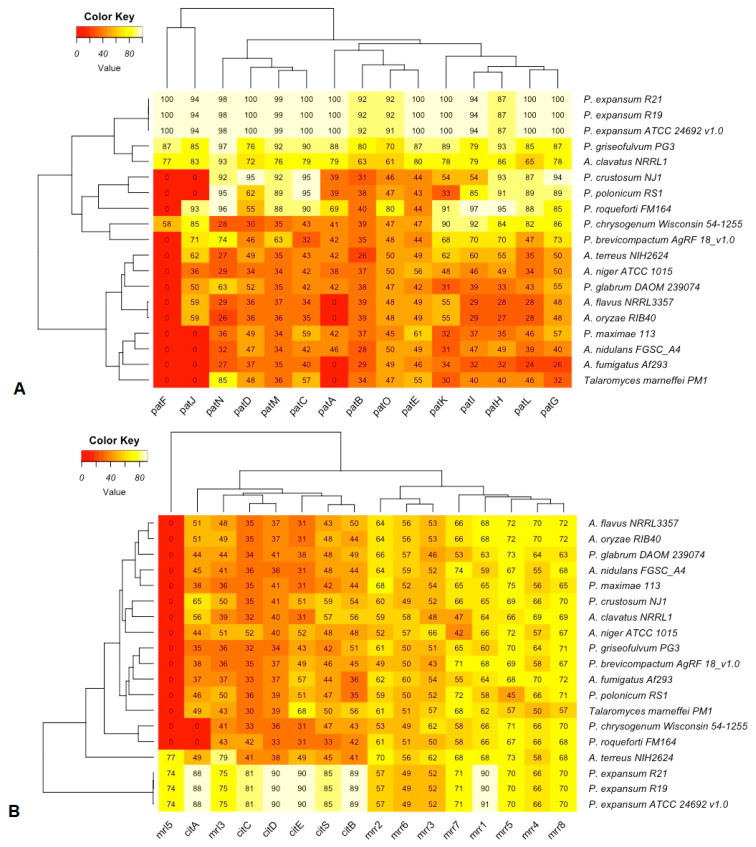
Functional conservation of patulin (**A**) and citrinin (**B**) gene clusters are estimated by TBLASTN identity analysis. Eleven *Penicillium* and seven *Aspergillus* strains were used.

Functional conservation of patulin and citrinin gene clusters from 12 *Penicillium* and 7 *Aspergillus* strains were estimated by identities calculated based on TBLASTN known protein sequences against genome sequences (Figure 4). *P*. *griseofulvum* PG3 was confirmed to produce patulin by HPLC analysis [21]. Patulin production by *P*. *expansum* R21 was carried out via TLC, and R19 was confirmed by TLC and GC-MS [64,65]. As the patulin biosynthesis gene cluster of *P*. *expansum* ATCC24692 strain is highly similar to those strains R21 and R19, this strain may also produce this mycotoxin. Even though all the members in the patulin gene cluster could be detected from *P*. *chrysogenum* Wisconsin 54-1255 and *A*. *clavatus* NRRL1, these strains did not produce patulin due to the lower identities of some genes such as *patD*, *patB,* and *patO* [66]. Furthermore, the *patF* gene cannot be detected from most *Aspergillus* strains and the other seven *Penicillium* strains. Both RS1 and NJ1 strains also lacked the *patJ* gene and had low identity matches of the *patO*, *patE*, *patA*, *patK*, and *patB* genes (Figure 4A). The citrinin gene cluster could be detected from three *Penicillium* strains (*P*. *expansum* R19, R21, and ATCC24692) and one *Aspergillus* strain (*A*. *terreus* NIH2624) but cannot be detected from the other strains (lacking *mrl5* gene). For all the three *P. expansum* strains (R19, R21, and ATCC24692), *mrr2*, *mrr6*, and *mrr3* had lower than 60% identities; for the *A*. *terreus* NIH2624 strain, six genes in citrinin gene cluster (*citA*, *citC*, *citD*, *citE*, *citS*, and *citB*) had lower than 50% identities (Figure 4B).

Altogether, 59, 52, and 39 secondary metabolite gene clusters were found in strains R21, NJ1, and 113, respectively. The key enzymes of fungal secondary metabolite biosynthesis are non-ribosomal peptide synthetases (NRPS) and polyketide synthases (PKS). The NRPS and T1PKS secondary metabolite gene clusters with predicted core structures from these three *Penicillium* strains are shown in Table S4. *P*. *polonicum* and *P*. *crustosum* (7 in RS1 and 6 in NJ1 strains) had the highest numbers of gene clusters predicted for terpene synthesis; for *P. expansum* R19 and R21, and *P*. *maximae* 113, each had two gene clusters predicted for terpene synthesis (Table S5).

### 3.5. Effects of VOCs Emitted from Penicillium Species on the Third Instar Drosophila Larvae

**Figure 5:**
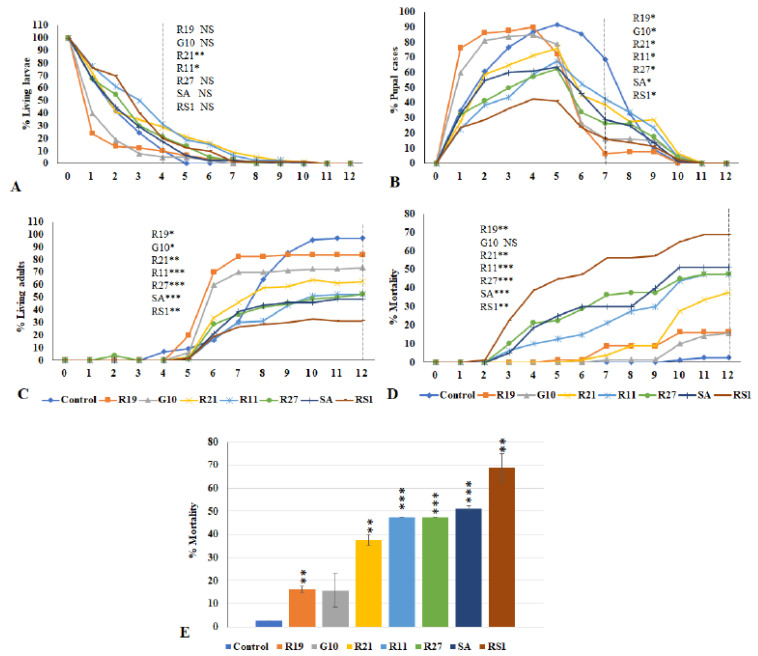
Development and viability of *Drosophila* larvae exposed to VOCs emitted by *Penicillium* species. The percentages of living larvae (**A**), pupae (**B**), living adults (**C**), and dead flies (**D**,**E**) are shown. Asterisks (*) indicate significant difference from control (* *p* < 0.05, ** *p* < 0.005, *** *p* < 0.0005). NS denotes not significant, and N = 90. Dashed vertical lines denote the days for statistical analyses. R19, R21, R11, and R27 are *P*. *expansum* species isolated from apple; *P. crustosum* G10 and *P. polonicum* RS1, *P*. *solitum* SA are species isolated from apple and peach seed, respectively.

To evaluate the effects of VOCs emitted from seven *Penicillium* strains, the numbers of larvae (Figure 5A), pupae (Figure 5B), adults (Figure 5C), and dead flies (Figure 5D) were counted daily for 12 days. Data are calculated as percentages of a given metamorphic stage during the *Drosophila* metamorphic cycle. Some flies never grew into adults; thus, the number of dead flies includes both dead larvae and dead pupae, which reflects the percent toxicity from exposure to the *Penicillium* VOCs mixtures (Figure 5E). Usually, control larvae pupate after 4 days; however, significant delays in pupation were observed for larvae exposed to VOCs emitted from *Penicillium* strains R21 (*p* < 0.005) and R11 (*p* < 0.05) (Figure 5A). By the 7th day, exposure to VOCs from all tested strains caused significant delays in pupation. Exposure to VOCs from strains R19 and G10 appeared to induce quicker transition to adulthood for surviving flies (Figure 5B). By the 10th to 12th day, 95–97% of the pupae from the controls had emerged as adults. The living adult percentages of flies exposed to VOCs from R19, G10, R21, R11, R27, SA, and RS1 by the 12th day were 84%, 74%, 63%, 53%, 53%, 49%, and 31%, respectively (Figure 5C). VOCs from *P*. *solitum* SA and *P*. *polonicum* RS1 were the most toxic, killing over 50% of the larvae; while *P*. *expansum* R19 and *P. crustosum* G10 were the least toxic, with an observed mortality of about 15% (Figure 5D,E). In summary, exposure of *Drosophila* larvae to VOCs emitted by *Penicillium* spp. VOCs negatively affected fly development and viability.

### 3.6. Identification of Putative Lipoxygenase Genes

**Figure 6:**
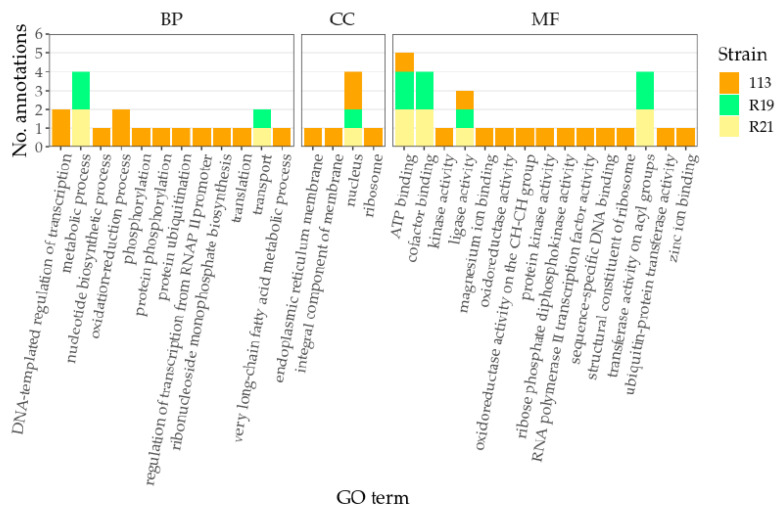
GO annotation frequencies of genes surrounding putative lipoxygenase genes. Two sets of genetic neighbors were inspected for 113, compared to only one set for strains R19 and R21 each. Panels are labeled by GO parent terms: BP, biological process; CC, cellular component; MF, molecular function.

**Figure 7:**
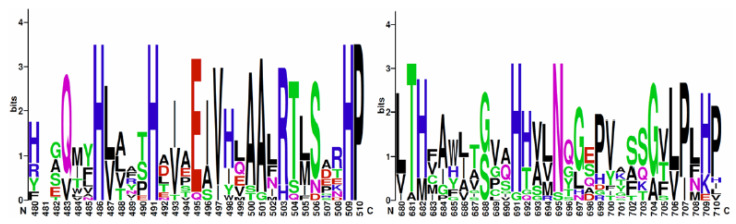
Sequence logos of putative metal-ligand binding sites of manganese lipoxygenases. Using the known structure of manganese lipoxygenase from *Magnaporthe oryzae* [67] as a guide, the metal-ligand binding residues in the multiple sequence alignment are located at H486, H491, H691, and N695. The three manganese lipoxygenase proteins identified in this study had the same residues. The sequence XM_007913037.1 from *Togninia minima* had a glycine and a serine at positions 691 and 695, respectively. The images were generated using WebLogo [68]. Colors represent chemical properties of amino acids: polar, green; basic, blue; acidic, red; hydrophobic, black; glutamine and asparagine are pink.

A total of 46 fungal lipoxygenase protein sequences were retrieved from GenBank, 15 of which were specifically annotated as manganese lipoxygenases (Table S6). Probing for a biosynthetic operon, genes surrounding the lipoxygenase genes were functionally annotated. One manganese lipoxygenase gene was identified in strains *P*. *maximae* 113 and *P*. *expansum* R19 and R21, none of which appeared to be within a biosynthetic operon. Any similarities were likely evidence of orthologous genome architecture, especially between *P*. *expansum* strains R19 and R21. Around the lipoxygenase genes, functional annotations ATP binding function and nuclear localization were the most abundant (Figure 6). Other *P*. *expansum*, *P*. *maximae,* and *P*. *crustosum* sequences found to be similar to lipoxygenases were annotated as cytochrome P450s, Golgi SNAP receptors, or hypothetical proteins (Table S7). Multiple sequence alignments of manganese lipoxygenases revealed conserved amino acid residues within the sequences that were also conserved in the three putative lipoxygenase genes identified in this study (Figure 7).

## 4. Discussion

Genomic and transcriptomic analyses of *Penicillium* species have revealed several interesting genes and mechanisms likely involved in the adaption of this species to various environments [24]. The *Penicillium* genomes previously studied have estimated genome sizes, ranging from 25 Mb to 36 Mb, and a low proportion of interspersed repeats, ranging from 0.32 to 1.71%. The genomes of the three *Penicillium* strains studied here are consistent with the previous studies [24]. A direct relationship between host range and genome size has been proposed for fruit pathogens [13,17]. Compared to the genome sizes of *P*. *digitatum* (~25.7 Mb in average, only infects citrus fruits), *P*. *italicum* (~29 Mb, intermediate host range, mainly infects citrus fruits), and *P*. *expansum* (~31 Mb, infects pome and stone fruits), the genome sizes of *P*. *expansum* R21 (~34 Mb) and *P*. *maximae* strain 113 (~35 Mb) are fairly large. *Penicillium* species usually have four to eight chromosomes [69,70], and it is ideal to assemble raw sequencing data into complete individual chromosomes. Unfortunately, no complete reference *P*. *maximae* genome is available, so we performed a de novo assembly of strain 113. In the future, complementary genomic sequencing methods such as PacBio or other long-read sequencing may help assemble a more accurate genome [71,72].

The Food and Agriculture Organization of the United Nations has found that about one-fourth of the world’s food crops are contaminated with mycotoxins every year [73,74]. *Penicillium* produces a wide range of mycotoxins of which the most important are patulin, citrinin, and ochratoxin A [75,76]. Patulin, a potential carcinogen [36], is mainly produced by strains of *P*. *expansum* [13]. The patulin gene cluster in *P. expansum* consists of fifteen genes in the following order: *patH*, *patG*, *patF*, *patE*, *patD*, *patC*, *patB*, *patA*, *patM*, *patN*, *patO*, *patL*, *patI*, *patJ*, and *patK* [28,77]. The transcription factor *patL* and the *patK* gene coding the backbone enzyme are present in all analyzed strains but with diverse similarities (24–100% for *patL* and 30–100% for *patK*, Figure 4A) indicating that these two genes experienced different evolutionary histories during adaptation to different environments and hosts. In this study, the complete and functional patulin gene cluster can be detected from three strains of *P*. *expansum* (R21, R19, and ATCC24692). We confirmed the production of patulin from two of these strains. No patulin is produced by *P*. *chrysogenum* Wisconsin 54-1255 or *A*. *clavatus* NRRL1, which reflects the lower sequence identities of genes such as *patD*, *patB*, *patO, patH*, and *patL*. In the future, more strains from *A*. *clavatus* should be tested as it is a reported patulin producer [66].

Citrinin, a nephrotoxic mycotoxin, was first isolated from *P*. *citrinum* and is a less toxic substance than patulin [78]. Sixteen putative genes: *pksCT*, *ctnI*, *orf3*, *ctnE*, *orf1*, *ctnH*, *ctnD*, *orf4*, *ctnA*, *ctnR*, *orf5*, *orf6*, *orf7*, and *orf8*, may be involved in citrinin biosynthesis [79,80]. The complete citrinin gene cluster has been identified from three *Penicillium* strains (*P*. *expansum* R19, R21, and ATCC24692) and one *Aspergillus* strain (*A*. *terreus* NIH2624). Our analysis indicates that the *citS* gene is present in all the analyzed strains with identities from 33% to 85% (Figure 4B). One recent study suggests that it could be an essential gene in *Penicillium steckii* [81]. The *mrl3/ctnA* (*P*. *citrinum*/*P*. *expansum*) sequence is also present in the analyzed strains (identities from 36% to 79%, Figure 4B) and may be similar to the specific transcriptional factor *patL* gene, which is often located inside the toxin cluster and activates all the genes in the cluster [82]. We did not verify citrinin production in any strain. Complete patulin and citrinin gene clusters could not be identified from *P*. *maximae* 113. In summary, patulin and citrinin are mainly produced by strains of *P*. *expansum*. Our patulin gene cluster identity and analysis support previous predictions that the cluster in *P*. *griseofulvum* PG3 (lower identity) is likely an ancestral trait in *Penicillium* spp. (higher identities), predating the divergence of *P*. *roqueforti* FM164 (lacking the *patF* gene) [22].

VOCs are chemically diverse groups of low-molecular-weight compounds that evaporate easily at normal temperatures and serve biologically targeted and diverse functions as semiochemicals, antimicrobials, and defense response chemicals [83,84]. Eight carbon compounds such as 1-octen-3-ol, 3-octanol, and 3-octanone are among the most common fungal VOCs [39]. In previous studies, low concentrations of the vapor form of several eight carbon compounds including 1-octen-3-ol are toxic to *Drosophila* larvae and adult flies [41,85,86]. 1-Octen-3-ol selectively affects dopaminergic neurons in adult *Drosophila* brain and induces Parkinson’s-like behavioral alterations in a fly model for this disease [87,88,89]. Moreover, we showed that the R and S enantiomers of 1-octen-3-ol have differential effects on the transcriptome of *P*. *chrysogenum* [90]. We found that the VOCs emitted by *Penicillium maximae* 113 caused a relatively high toxicity (60%) in the *Drosophila* larvae toxicity assay. Solid-phase microextraction gas chromatography mass spectrometry analysis revealed that a higher diversity and concentration (up to 300 ng/L) of VOCs were emitted by this strain after longer periods of cultivation (18 days compared to 7 days) [14].

We also tested the toxic effects of VOCs from several *Penicillium* strains; our data revealed that VOCs from *P*. *polonicum* strains are more toxic than those emitted by the other strains (Figure 5). More research, including GC-MS analyses, to elucidate individual VOC profiles of different species are needed to better understand their toxic effects. The group of fatty acid dioxygenases called Ppos (psi (precocious sexual inducer)-producing oxygenases) and lipoxygenases (LOXs) are candidates for the enzymes involved in eight carbon volatile formation in fungi [91]. In this study, putative lipoxygenase genes that would contribute to production of VOCs were identified in *Penicillium* *maximae* strain 113 and *P*. *expansum* strains R19 and R21. We will clone these candidate genes to explore their functions in our future work.

In summary, our analyses showed that different *P*. *expansum* strains have different secondary metabolite gene clusters, and their numbers are very changeable within this species. Comparative genomic studies have revealed that there are interesting patterns of gains or losses of genes involved in the production of secondary metabolites that act as effectors in pathogens [92]. Horizontal gene transfer is an ongoing and successful ecological strategy for many fungal species to acquire the novel secondary metabolic gene clusters [5,93] and may be occurring in mycotoxigenic species of *Penicillium*. However, this hypothesis needs further experimental verification.

## Figures and Tables

**Table 1:** The assembly statistics and gene predictions.

*Penicillium* spp. Assembly	R21	NJ1	113
**No. of all scaffolds**	257	91	228
**Bases in all scaffolds (bp)**	35,046,069	31,778,960	34,025,097
**No. of large scaffolds (>1000 bp)**	244	48	135
**Bases in large scaffolds (>1000 bp)**	35,037,781	31,755,870	33,977,500
**Largest length (bp)**	1,693,895	5,141,995	4,666,273
**Scaffold N50 (bp)**	377,946	3,327,763	1,432,551
**Scaffold N90 (bp)**	791,867	1,217,655	346,259
**GC content**	48.23%	47.95%	48.63%
**N rate**	0.53%	0.81%	1.33%
**No. of all contigs**	573	954	737
**Bases in all contigs (bp)**	34,858,998	31,522,745	33,572,562
**No. of large contigs (>1000 bp)**	556	712	511
**Bases in large contigs (bp)**	34,847,611	31,399,673	33,460,720
**Length of largest contig (bp)**	556,579	346,776	614,748
**Contig N50 (bp)**	108,376	93,897	157,787
**Contig N90 (bp)**	35,359	26,540	39,668
***Penicillium* spp. gene prediction**			
**No. genes**	12,707	12,057	12,649
**Gene total length (bp)**	18,790,875	17,215,819	17,946,096
**Gene density (genes per kb)**	0.362	0.379	0.371
**Gene average length**	1478	1427	1418
**GC content in gene region (%)**	52.3	52.2	52.4
**Gene/genome (%)**	53.6	54.2	52.7
**Intergenetic region length (bp)**	16,255,194	14,563,141	16,079,001
**GC content in intergenetic region (%)**	43.4	43.0	44.4
**Intergenetic length/genome (%)**	46.4	45.8	47.3

## Supplementary Materials

The following are available online at https://www.mdpi.com/article/10.3390/jof7090743/s1, Figure S1: COG analyses and Go annotation of three Penicillium species, Table S1: Sequencing data statistical analysis, Table S2: 17-mer analysis, Table S3: Repeated sequence analysis, Table S4: NRPS and T1PKS secondary metabolite gene clusters, Table S5: Secondary metabolite gene clusters predicted for terpene synthesis in Penicillium species, Table S6: Putative lipoxygenase genes returned by HMMER searches and their GO biological processes and molecular functions terms, Table S7: Lipoxygenase amino acid sequences retrieved from NCBI GenBank.
