# Supplementary material for: Genomic Analyses of Penicillium Species Have Revealed Patulin and Citrinin Gene Clusters and Novel Loci Involved in Oxylipin Production
Source: J Fungi (Basel). 2021 Sep 9;7(9):743. doi: 10.3390/jof7090743 (PMC8464941; doi:10.3390/jof7090743)
Supplement: Supplementary file 1 [file jof-07-00743-s001.zip › jof-1341125-supplementary.pdf]

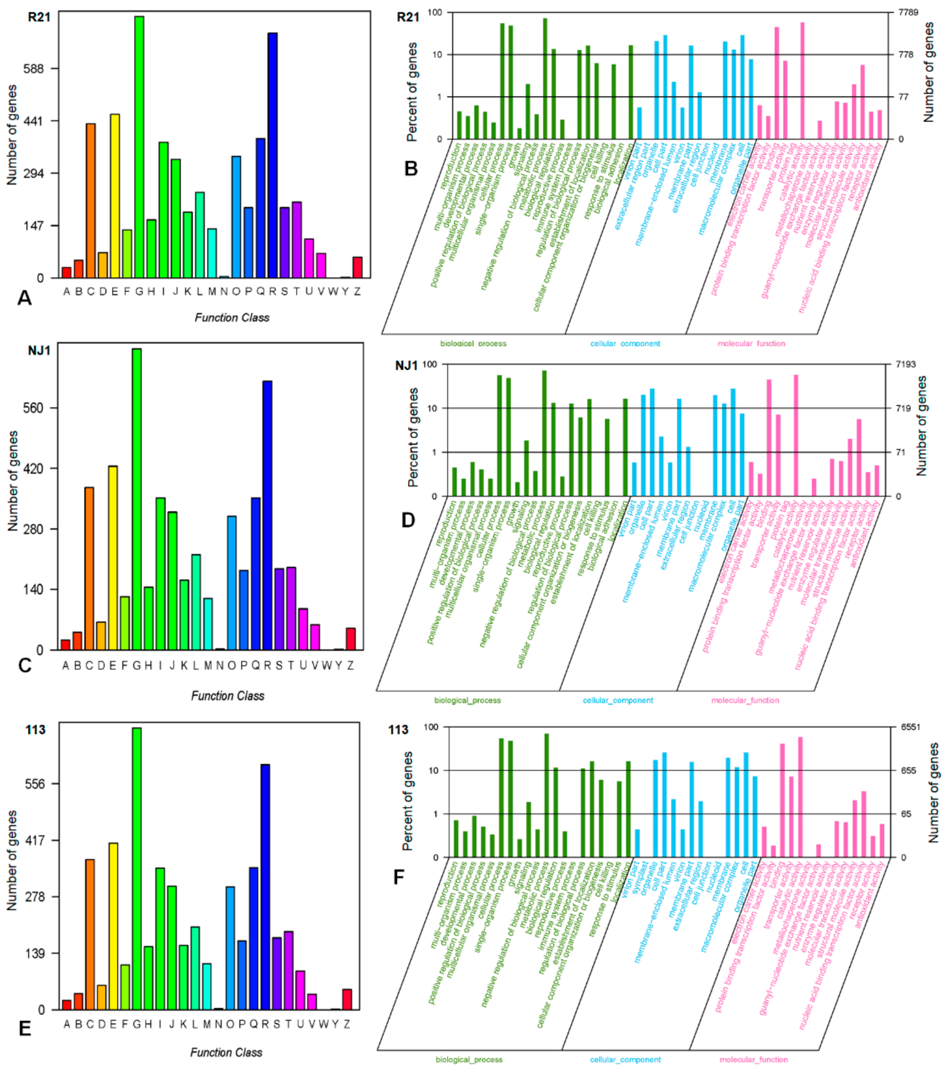
R21
Number of genes
588
441
294
147
0
A
A B C D E F G H I J K L M N O P Q R S T U V W Y Z
Function Class
R21
Percent of genes
Number of genes
100
10
1
0
7789
778
77
0
B
biological_process
cellular_component
molecular_function
NJ1
Number of genes
560
420
280
140
0
C
A B C D E F G H I J K L M N O P Q R S T U V W Y Z
Function Class
NJ1
Percent of genes
Number of genes
7193
719
71
D
biological_process
cellular_component
molecular_function
113
Number of genes
556
417
278
139
0
E
A B C D E F G H I J K L M N O P Q R S T U V W Y Z
Function Class
113
Percent of genes
Number of genes
6551
655
65
F
biological_process
cellular_component
molecular_function

Figure S1: COG analyses and Go annotation of three *Penicillium* species. COG analysis and GO annotation of R21 are shown in (**A**) and (**B**); COG analysis and GO annotation of NJ1 are shown in (**C**) and (**D**); COG analysis and GO annotation of 113 are shown in (**E**) and (**F**), respectively. Function classes are as follows, A: RNA processing and modification; B: Chromatin structure and dynamics; C: Energy production and conversion; D: Cell cycle control, cell division, chromosome partitioning; E: Amino acid transport and metabolism; F: Nucleotide transport and metabolism; G: Carbohydrate transport and metabolism; H: Coenzyme transport and metabolism; I: Lipid transport and metabolism; J: Translation, ribosomal structure and biogenesis; K: Transcription; L: Replication, recombination and repair; M: Cell wall/membrane/envelope biogenesis; N: Cell motility; O: Posttranslational modification, protein turnover, chaperones; P: Inorganic ion transport and metabolism; Q: Secondary metabolites biosynthesis, transport and catabolism; R: General function prediction only; S: Function unknown; T: Signal transduction mechanisms; U: Intracellular trafficking, secretion, and vesicular transport; V: Defense mechanisms; W: Extracellular structures; Y: Nuclear structure; Z: Cytoskeleton.

Supplementary Tables

Table S1. Sequencing data statistical analysis.

**Sample**	**Raw sequencing data statistics**						**High quality data statistics**			
	**Insert Size (bp)**	**Read Length (bp)**	**No. Paired Reads**	**Total Bases (bp)**	**≥ Q20 (%)**	**Average Depth**	**No. Paired Reads**	**No. Single Reads**	**Total Bases (bp)**	**Average Depth**
R21_PE	410	150	12,231,104	3,669,331,200	96.58	105X	11,800,356	412,156	3,519,910,684	101X
R21_2K	2k	150	12,719,398	3,815,819,400	94.48	109X	11,731,645	453,821	3,393,051,185	97X
R21_8K	8k	150	9,766,483	2,929,944,900	93.90	84X	8,656,030	394,052	2,524,221,021	72X
NJ1_PE	450	150	23,457,645	7,084,208,790	87.03	222X	21,875,156	1,548,964	6,033,645,654	189X
NJ1_2K	2k	150	11,620,194	3,486,058,200	94.84	109X	11,123,774	418,406	3,261,701,253	102X
NJ1_8K	8k	150	12,839,765	3,851,929,500	94.49	120X	12,201,260	435,339	3,580,026,788	112X
113_PE	500	150	20,930,298	6,320,949,996	89.34	186X	19,701,019	1,217,922	5,577,148,842	164X
113_2K	2k	150	10,603,360	3,202,214,720	90.95	94X	10,230,196	354,852	2,860,019,724	84X
113_8K	8k	150	7,568,123*2	2,285,573,146	86.58	67X	7,221,663*2	325,535	1,924,500,423	57X

Table S2. 17-mer analysis.

K-mer	K-mer_num	Pk_depth	Genome size (bp)	No. used_bases	No. used_reads
R21-17	2,054,735,844	6	342,455,974	2,446,114,100	24,461,141
NJ1-17	3,935,966,496	121	32,528,648	4,685,674,400	46,856,744
113-17	3,164,741,162	90	35,163,791	3,794,856,730	39,382,223

Table S3. Repeated sequence analysis.

**Strains**	**R21**		**NJ1**		**113**	
**Type**	**Length (bp)**	**% Genome**	**Length (bp)**	**% Genome**	**Length (bp)**	**% Genome**
SINEs	1,191	0.00	1,729	0.01	1,176	0.00
LINEs	12,381	0.04	18,903	0.06	20,525	0.06
LTR elements	155	0.00	62	0.00	32	0.00
DNA elements	2,827	0.01	1,816	0.01	1,854	0.01
Unclassified	360	0.00	-	-	-	-
Total length	262,677	0.80	365,865	1.15	279,139	0.82

Table S4. NRPS and T1PKS secondary metabolite gene clusters.

Strains	Types	Locations	Predicted core structures
**R21-gene cluster 1 (scaffold2)**<br><br>**113-gene cluster 31 (scaffold 25)**	NRPS	211595 - 264273 nt for cluster 1 in R21;<br><br>224915 - 271947 nt for cluster 31 in 113	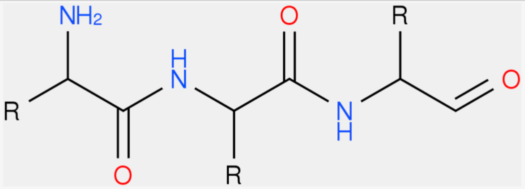
**R21-gene cluster 3 (scaffold 4)**	NRPS	377720 - 434246 nt	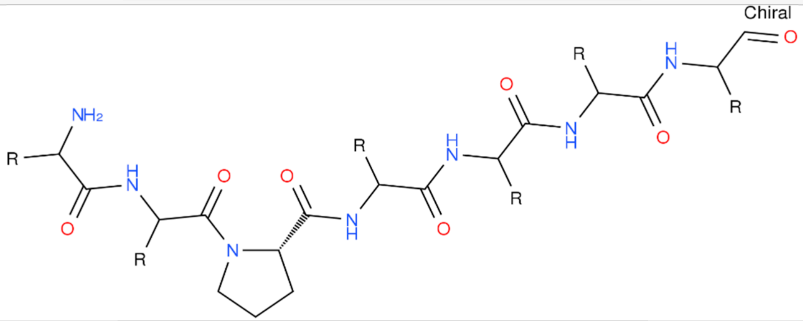
**R21-gene cluster 7 (scaffold 6)**	NRPS	245141 - 291497 nt	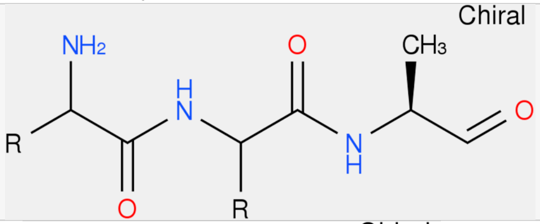
**R21-gene cluster 8 (scaffold 10)**	NRPS	56914 - 112491 nt	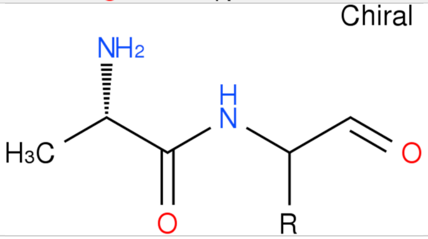

**R21-gene cluster 11 (scaffold 11)**	NRPS	81347 - 131066 nt	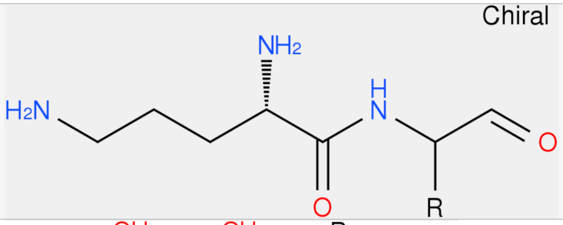
**R21-gene cluster 18 (scaffolder 18)**	NRPS	285774 - 331605 nt	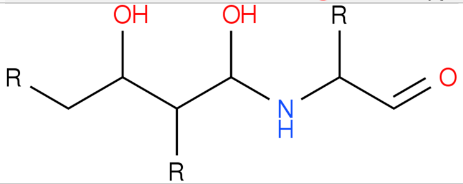
**R21-gene cluster 19 (scaffold 18)**<br><br>**NJ1-gene cluster 7 (scaffold 1)**<br><br>**NJ1-gene cluster 22 (scaffold 3)**<br><br>**NJ1-gene cluster 25 (scaffold 4)**<br><br>**NJ1-gene cluster 29 (scaffold 4)**<br><br>**NJ1-gene cluster 30 (scaffold 4)**<br><br>**NJ1-gene cluster 51 (scaffold 13)**	NRPS<br><br>NRPS<br><br>NRPS<br><br>Indole-NRPS<br><br>NRPS<br><br>NRPS<br><br>NRPS	805936 - 848483 nt for cluster 19 in R21;<br><br>2658543 - 2715172 nt for cluster 7 in NJ1;<br><br>3207632 - 3254759 nt for cluster 22 in NJ1;<br><br>342701 - 385365 nt for cluster 25 in NJ1;<br><br>2882322 - 2936673 nt for cluster 29 in NJ1<br><br>2920715 - 2969639 nt for cluster 30 in NJ1;<br><br>64947 - 117640 nt for cluster 51 in NJ1	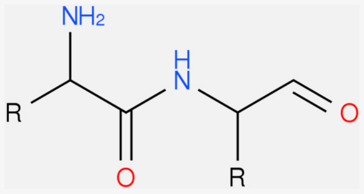

**R21-gene cluster 22 (scaffold 26)**	NRPS	180328 - 226864 nt	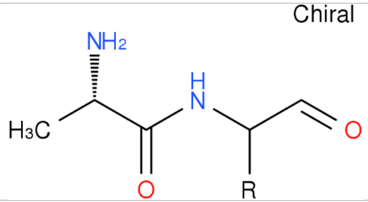
**R21-gene cluster 27 (scaffold 34)**<br><br>**NJ1-gene cluster 15 (scaffold 2)**<br><br>**NJ1-gene cluster 21 (scaffold 3)**	T1PKS-indole<br><br>NRPS-T1PKS<br><br>T1PKS	234250 - 284037 nt for cluster 27 in R21;<br><br>3156920 - 3217231 nt for cluster 15 in NJ1;<br><br>1759453 - 1822403 nt for cluster 21 in NJ1	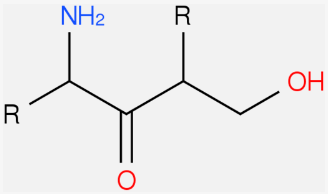
**R21-gene cluster 28 (scaffold 34)**	Indole-NRPS	342325 - 385818 nt	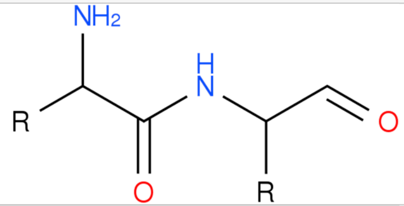
**R21-gene cluster 29 (scaffold 36)**	NRPS	216912 - 261666 nt	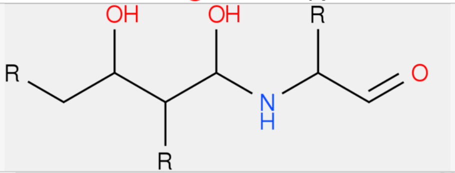
**R21-gene cluster 30 (scaffold 39)**	NRPS-lantipeptide-T1PKS	398514 - 462608 nt	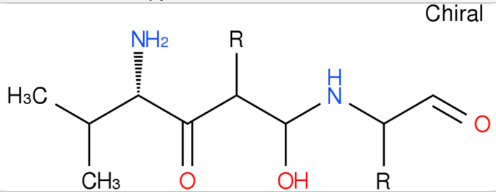

**R21-gene cluster 34 (scaffold 42)**	T1PKS	82889 - 144367 nt	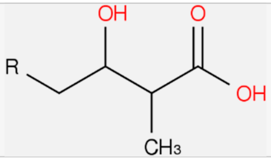
**R21-gene cluster 38 (scaffold 55)**<br><br>**NJ1-gene cluster 32 (scaffold 5)**	NRPS	89344 - 136024 nt for cluster 38 in R21;<br><br>1179369 - 1225774 nt for cluster 32 in NJ1	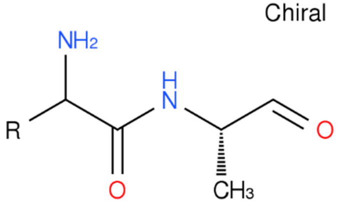
**R21-gene cluster 39 (scaffold 56)**<br><br>**NJ1-gene cluster 13 (scaffold 2)**<br><br>**NJ1-gene cluster 27 (scaffold 4)**<br><br>**NJ1-gene cluster 40 (scaffold 8)**<br><br>**113-gene cluster 7 (scaffold 3)**<br><br>**113-gene cluster 38 (scaffold 56**	NRPS-T1PKS<br><br>NRPS-T1PKS<br><br>T1PKS<br><br>NRPS-T1PKS<br><br>NRPS<br><br>T1PKS-NRPS	55063 - 107260 nt for cluster 39 in R21;<br><br>2407682 - 2471509 nt for cluster 13 in NJ1;<br><br>714469 - 779347 nt for cluster 27 in NJ1;<br><br>5744 - 57179 nt for cluster 40 in NJ1;<br><br>879987 - 924774 nt for cluster 7 in 113;<br><br>1 - 22358 nt for cluster 38 in 113	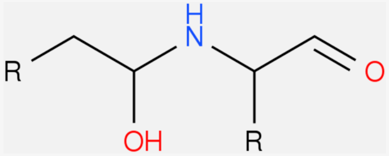
**R21-gene cluster 43 (scaffold 74)**	NRPS	34965 - 90439 nt	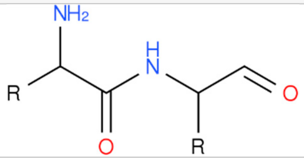

**R21-gene cluster 44 (scaffold 75)**	NRPS	23802 - 85362 nt	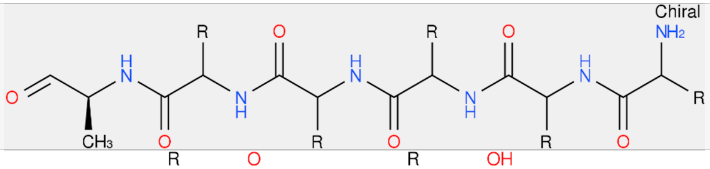
**R21-gene cluster 47 (scaffold 93)**	NRPS-T1PKS	1 - 61539 nt	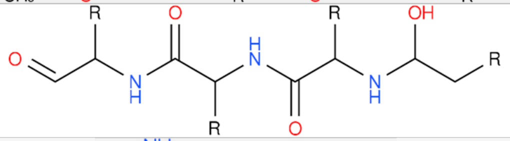
**R21-gene cluster 48 (scaffold 104)**	indole-NRPS	18195 - 61688 nt	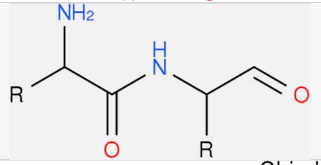
**R21-gene cluster 49 (scaffold 107)**	T1PKS-NRPS	8122 - 59405 nt	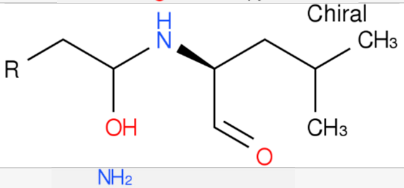
**R21-gene cluster 50 (scaffold 111)**	T1PKS	16706 - 71065 nt	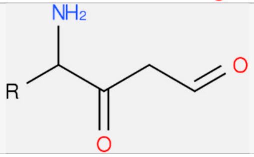
**R21-gene cluster 52 (scaffold 127)**<br><br>**NJ1-gene cluster 2 (scaffold 1)**<br><br>**113-gene cluster 15 (scaffold 6)**	NRPS	50265 - 107402 nt for cluster 52 in R21;<br><br>971605 - 1028764 nt for cluster 2 in NJ1;<br><br>838414 - 896403 nt for cluster 15 in 113	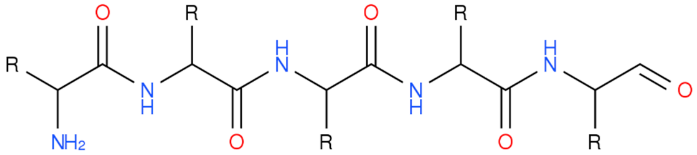

**R21-gene cluster 55 (scaffold 136)**<br><br>**113-gene cluster 20 (scaffold 11)**<br><br>**113-gene cluster 36 (scaffold 32)**	NRPS	1 - 31336 nt for cluster 55 in R21;<br><br>198448 - 248440 nt for cluster 20 in 113;<br><br>1 - 33075 nt for cluster 36 in 113	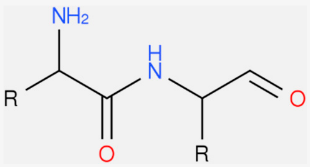
**NJ1-gene cluster 6 (scaffold 1);**<br><br>**NJ1-gene cluster 16 (scaffold 2)**	NRPS	1973959 - 2030489 nt for cluster 6 in NJ1;<br><br>3357491 - 3416063 nt for cluster 16 in NJ1	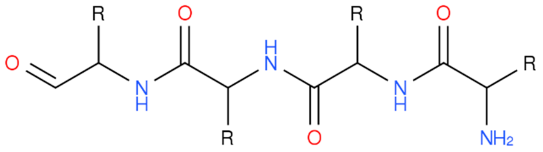
**NJ1-gene cluster 9 (scaffold 1)**	NRPS	4608499 - 4666142 nt	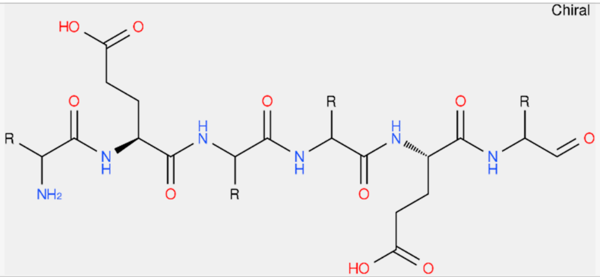
**NJ1-gene cluster 12 (scaffold 2)**	T1PKS-NRPS	1276282 - 1331278 nt	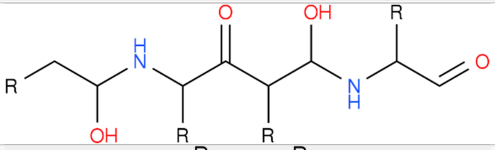
**NJ1-gene cluster 19 (scaffold 3)**	T1PKS	1295087 - 1339392 nt	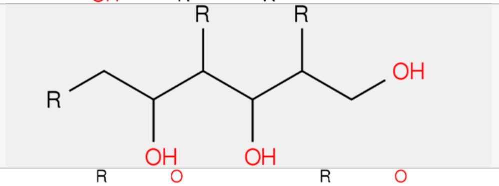
**NJ1-gene cluster 24 (scaffold 3)**	NRPS-T1PKS	3470394 - 3545313 nt	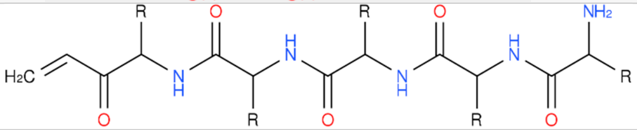

**NJ1-gene cluster 31**	NRPS-T1PKS	30706 - 95665 nt	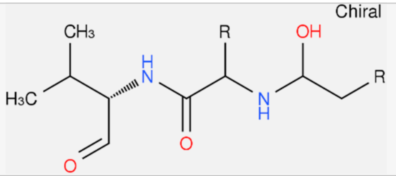
**NJ1-gene cluster 52 (scaffold 13)**	T1PKS	172207 - 215368 nt	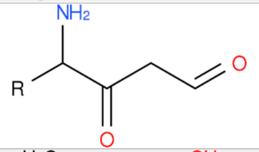
**113-gene cluster 21 (scaffold 13)**	T1PKS	525965 - 584277 nt	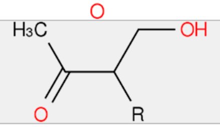
**113-gene cluster 30 (scaffold 24)**	NRPS	62223 - 117514 nt	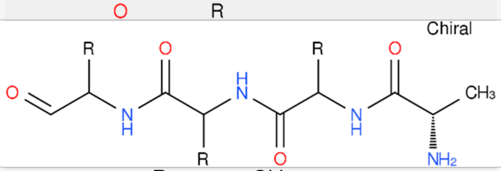
**113-gene cluster 33 (scaffold 26)**	T1PKS	273762 - 317862 nt	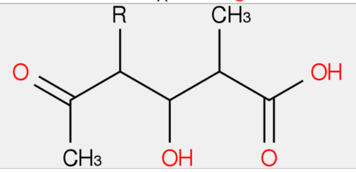

Note: NRPS: non ribosomal peptide synthetases; T1PKS: type 1 polyketide synthases.

Table S5. Secondary metabolite gene clusters predicted for terpene synthesis in *Penicillium* species.

Penicillium	**Strains**	**Locations**
P. expansum	R19-gene cluster 15 (c00211_gi_6078)	1 - 40394 nt
	R19-gene cluster 30 (c00498_gi_6078)	12560 - 42693 nt
	R21- gene cluster 45 (scaffold 81)	50802 - 72079 nt
	R21-gene cluster 51 (scaffold 116)	43276 - 65327 nt
P. polonicum	RS1-gene cluster 2 (c00001_gi_1024)	924501 - 945689 nt
	RS1-gene cluster 3 (c00001_gi_1024)	946852 - 967463 nt
	RS1-gene cluster 9 (c00002_gi_1024)	140922 - 162637 nt
	RS1-gene cluster 31 (c00002_gi_1024)	10651398 - 10672615 nt
	RS1-gene cluster 51 (c00007_gi_1024)	527429 - 548598 nt
	RS1-gene cluster 55 (c00007_gi_1024)	1813734 - 1835734 nt
	RS1-gene cluster 59 (c00007_gi_1024)	4135222 - 4156499 nt
P. crustosum	NJ1-gene cluster 18 (scaffold 2)	4398321 - 4420068 nt
	NJ1-gene cluster 37 (scaffold 6)	1586748 - 1607901 nt
	NJ1-gene cluster 45 (scaffold 10)	155277 - 175887 nt
	NJ1-gene cluster 46 (scaffold 10)	181014 - 202246 nt
	NJ1-gene cluster 47 (scaffold 10)	381826 - 402886 nt
	NJ1-gene cluster 50 (scaffold 12)	63618 - 84895 nt
P. maximae	113-gene cluster 9 (scaffold 4)	885912 - 907312 nt
	113-gene cluster 26 (scaffold 17)	566137 - 587123 nt

Table S6. Putative lipoxygenase genes returned by HMMER searches and their GO biological processes and molecular functions terms.

Protein ID	Descriptive name	Amino acid length	GO terms
113_3054	linoleate 10R-lipoxygenase	1110	• responsive to oxidative stress<br>• oxidation-reduction process<br>• cellular oxidant detoxification<br>• peroxidase activity<br>• iron ion binding<br>• oxidoreductase activity<br>• heme binding
113_5301	Golgi SNAP receptor subunit 1	226	• ER to Golgi vesicle-mediated transport<br>• vesicle fusion<br>• protein transport<br>• SNAP receptor activity
113_10665	psi-producing oxygenase A	1080	• responsive to oxidative stress<br>• oxidation-reduction process<br>• cellular oxidant detoxification<br>• peroxidase activity<br>• iron ion binding<br>• oxidoreductase activity<br>• heme binding
R19_154	lipoxygenase	752	• oxidation-reduction process<br>• oxidoreductase activity<br>• metal ion binding
R19_7323	cytochrome P450	1073	• responsive to oxidative stress<br>• oxidation-reduction process<br>• cellular oxidant detoxification<br>• peroxidase activity<br>• iron ion binding<br>• oxidoreductase activity<br>• heme binding
R19_11425	cytochrome P450	1121	• responsive to oxidative stress

			• oxidation-reduction process<br>• cellular oxidant detoxification<br>• peroxidase activity<br>• iron ion binding<br>• oxidoreductase activity<br>• heme binding
R21_6403	cytochrome P450	1073	• responsive to oxidative stress<br>• oxidation-reduction process<br>• cellular oxidant detoxification<br>• peroxidase activity<br>• iron ion binding<br>• oxidoreductase activity<br>• heme binding
R21_7950	cytochrome P450	1121	• responsive to oxidative stress<br>• oxidation-reduction process<br>• cellular oxidant detoxification<br>• peroxidase activity<br>• iron ion binding<br>• oxidoreductase activity<br>• heme binding
R21_8720	lipoxygenase	752	• oxidation-reduction process<br>• oxidoreductase activity<br>• metal ion binding
NJ1_8610	cytochrome P450	1073	• responsive to oxidative stress<br>• oxidation-reduction process<br>• cellular oxidant detoxification<br>• peroxidase activity<br>• iron ion binding<br>• oxidoreductase activity<br>• heme binding
NJ1_9064	cytochrome P450	1119	• responsive to oxidative stress<br>• oxidation-reduction process

			• cellular oxidant detoxification<br>• peroxidase activity<br>• iron ion binding<br>• oxidoreductase activity<br>• heme binding
RS1_7484	hypothetical protein	610	• oxidation-reduction process<br>• oxidoreductase activity<br>• metal ion binding
RS1_7485	hypothetical protein	113	• oxidation-reduction process<br>• oxidoreductase activity<br>• metal ion binding
RS1_8523	cytochrome P450	1123	• responsive to oxidative stress<br>• oxidation-reduction process<br>• cellular oxidant detoxification<br>• peroxidase activity<br>• iron ion binding<br>• oxidoreductase activity<br>• heme binding
RS1_11455	cytochrome P450	1073	• responsive to oxidative stress<br>• oxidation-reduction process<br>• cellular oxidant detoxification<br>• peroxidase activity<br>• iron ion binding<br>• oxidoreductase activity<br>• heme binding

Note: Proteins that were identified as similar to manganese lipoxygenases are highlighted in yellow.

Table S7. Lipoxygenase amino acid sequences retrieved from NCBI GenBank.

Species and strain	Descriptive name	NCBI accessions
Aspergillus flavus	putative lipoxygenase gene, complete cds	EU486993.1
Aspergillus fumigatus Af293	arachidonate 5-lipoxygenase, putative (AFUA_4G02770), partial mRNA	XM_741370.1
Aspergillus fumigatus Af293	lipoxygenase (AFUA_7G00860), partial mRNA	XM_741751.1
Aspergillus ochraceus	lipoxygenase (lox) gene, partial cds	DQ087531.1
Aspergillus oryzae RIB40	arachidonate 5-lipoxygenase, mRNA	XM_001822113.2
Aureobasidium namibiae CBS 147.97	lipoxygenase partial mRNA	XM_013571613.1
Chaetomium thermophilum var. thermophilum DSM 1495	lipoxygenase-like protein	XM_006697090.1
Colletotrichum fioriniae PJ7	manganese lipoxygenase mRNA	XM_007598573.1
Colletotrichum gloeosporioides Nara gc5	arachidonate 8-lipoxygenase (CGGC5_5785), partial mRNA	XM_007276522.1
Colletotrichum gloeosporioides Nara gc5	lipoxygenase 1 (CGGC5_3431), partial mRNA	XM_007273701.1
Eutypa lata UCREL1	putative manganese lipoxygenase protein mRNA	XM_007789827.1
Eutypa lata UCREL1	putative manganese lipoxygenase protein mRNA	XM_007790759.1
Eutypa lata UCREL1	putative manganese lipoxygenase protein mRNA	XM_007798792.1
Gaeumannomyces graminis var. avenae strain CBS 870.03	manganese lipoxygenase gene, complete cds	AY040824.2
Gaeumannomyces graminis var. tritici	manganese lipoxygenase mRNA, partial cds	AY040825.1
Gaeumannomyces graminis var. tritici R3-111a-1	manganese lipoxygenase mRNA	XM_009223395.1
Glarea lozoyensis ATCC 20868	lipoxygenase mRNA	XM_008081989.1
Gibberella moniliformis	lipoxygenase (LOX1) gene, complete cds	AY699875.1
Grosmannia clavigera kw1407	arachidonate 8-lipoxygenase partial mRNA	XM_014315415.1
Grosmannia clavigera kw1407	arachidonate 15-lipoxygenase precursor partial mRNA	XM_014316099.1
Isaria fumosorosea ARSEF 2679	lipoxygenase partial mRNA	XM_018852750.1
Magnaporthe grisea	manganese lipoxygenase mRNA, partial cds	AY829440.1
Magnaporthe grisea	manganese lipoxygenase mRNA, partial cds	AY858988.1
Magnaporthe oryzae	manganese lipoxygenase	KT734829.1
Magnaporthe oryzae 70-15	manganese lipoxygenase partial mRNA	XM_003715958.1
Metarhizium acridum CQMa 102	putative lipoxygenase 1 partial mRNA	XM_007817565.1
Marssonina brunnea f. sp. 'multigermtubi' MB_m1	manganese lipoxygenase (MBM_04109), mRNA	XM_007291936.1
Metarhizium acridum CQMa 102	lipoxygenase partial mRNA	XM_007809403.1
Metarhizium brunneum ARSEF 3297	lipoxygenase partial mRNA	XM_014692946.1
Metarhizium majus ARSEF 297	lipoxygenase 1 partial mRNA	XM_014722715.1

Metarhizium majus ARSEF 297	lipoxygenase 1 partial mRNA	XM_014725819.1
Metarhizium majus ARSEF 3297	lipoxygenase 1 partial mRNA	XM_014694440.1
Metarhizium robertsii ARSEF 23	lipoxygenase mRNA	XM_007819258.1
Metarhizium robertsii ARSEF 23	lipoxygenase mRNA	XM_007824276.1
Neosartorya fischeri NRRL 181	lipoxygenase, putative (NFIA_030810) partial mRNA	XM_001262544.1
Neosartorya fischeri NRRL 181	lipoxygenase, putative (NFIA_113540) partial mRNA	XM_001262719.1
Neurospora crassa OR74A	lipoxygenase mRNA	XM_953084.2
Paraphaeosphaeria sporulosa	lipoxygenase partial mRNA	XM_018181084.1
Paraphaeosphaeria sporulosa	lipoxygenase partial mRNA	XM_018184567.1
Penicillium expansum	lipoxygenase mRNA	XM_016740027.1
Phialocephala scopiformis	lipoxygenase partial mRNA	XM_018218786.1
Phialocephala scopiformis	lipoxygenase partial mRNA	XM_018220843.1
Pochonia chlamydosporia 170	manganese lipoxygenase protein partial mRNA	XM_018282120.1
Scedosporium apiospermum	linoleate 8R-lipoxygenase partial mRNA	XM_016789794.1
Togninia minima UCRPA7	putative manganese lipoxygenase protein mRNA	XM_007913037.1
Trichoderma gamsii	manganese lipoxygenase mRNA	XM_018802094.1
